# Antimicrobial usage and resistance in beef production

**DOI:** 10.1186/s40104-016-0127-3

**Published:** 2016-12-12

**Authors:** Andrew Cameron, Tim A. McAllister

**Affiliations:** 1 Faculty of Veterinary Medicine, University of Calgary, Calgary, AB Canada; 2 Agriculture and Agri-Food Canada, Lethbridge, AB Canada

**Keywords:** Antibiotics, Antimicrobial resistance, Antimicrobial usage, Beef production, Bovine pathogens, Bovine microbiota, Cattle, Enteropathogens, Fecal bacteria, Resistome

## Abstract

Antimicrobials are critical to contemporary high-intensity beef production. Many different antimicrobials are approved for beef cattle, and are used judiciously for animal welfare, and controversially, to promote growth and feed efficiency. Antimicrobial administration provides a powerful selective pressure that acts on the microbial community, selecting for resistance gene determinants and antimicrobial-resistant bacteria resident in the bovine flora. The bovine microbiota includes many harmless bacteria, but also opportunistic pathogens that may acquire and propagate resistance genes within the microbial community via horizontal gene transfer. Antimicrobial-resistant bovine pathogens can also complicate the prevention and treatment of infectious diseases in beef feedlots, threatening the efficiency of the beef production system. Likewise, the transmission of antimicrobial resistance genes to bovine-associated human pathogens is a potential public health concern. This review outlines current antimicrobial use practices pertaining to beef production, and explores the frequency of antimicrobial resistance in major bovine pathogens. The effect of antimicrobials on the composition of the bovine microbiota is examined, as are the effects on the beef production resistome. Antimicrobial resistance is further explored within the context of the wider beef production continuum, with emphasis on antimicrobial resistance genes in the food chain, and risk to the human population.

## Background

The emergence of antimicrobial resistance in bacterial pathogens is a serious global issue. Antimicrobial use in livestock, aquaculture, pets, crops, and humans selects for antimicrobial-resistant (AMR) bacteria that reside in agricultural and clinical biomes. Besides pathogens, AMR bacteria include many harmless and beneficial microbes acting as a genetic reservoir of AMR gene determinants (‘the resistome’ [1, 2]), which can be transferred via mechanisms of horizontal gene transfer (HGT) (reviewed in [3]) throughout the microbial community. With alarming frequency, untreatable human and animal pathogens with multiple AMR determinants arise. AMR in pathogens is commonly accepted as a result of widespread use and abuse of antimicrobials in agriculture and medicine. Although the use of antimicrobials in livestock and aquaculture has attracted particular attention, antimicrobials are also widely used in companion animals and in plant agriculture (e.g. oxytetracycline and streptomycin), for feed crops, and for tomatoes, citrus, and many other fruits [4]. Here, the focus is on large-scale beef production, where antimicrobials are routinely used to support animal welfare, and controversially, to promote growth and production efficiency. In this review, the usage of antimicrobials in cattle will be summarized along with recent studies on AMR explored within the context of the beef production system.

## Beef production

**Fig. 1:**
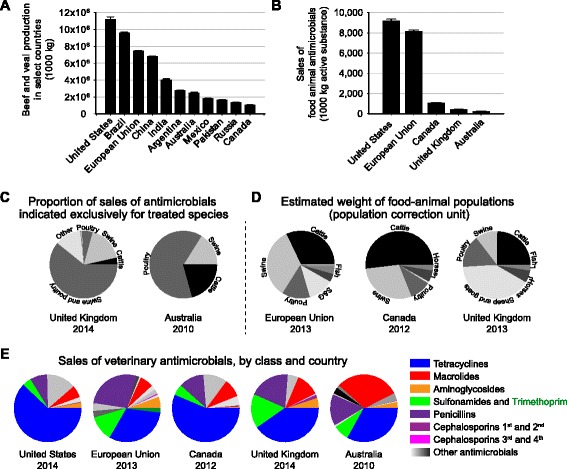
Major beef-producing countries and antimicrobial consumption. **a** Beef and veal production in select countries (t). Data from: ‘Livestock and Poultry: World Markets and Trade’. USDA. Foreign Agricultural Service [6]. **b** Antimicrobial sales, excluding ionophore sales, in reporting countries (t active substance). Data complied from multiple sources: [19–23] **c** Sales of antimicrobials authorised only for food‐producing animals, by species (t active substance) [22, 23]. **d** Weighted animal population (in PCU) [20, 21, 23]. **e** Proportion of sales of total antibiotic products by antimicrobial class (t active ingredient) [19–23]

Worldwide, beef production is the third largest meat industry (~65 million t globally), behind swine and poultry [5]. In 2015, the major beef producing countries included the United States (US) (11.4 million t), Brazil (9.6 million t), the 28 member countries of the European Union (EU) (7.5 million t), China (6.7 million t), and India (4.5 million t) (Fig. 1a) [6] with the global beef cattle population exceeding 1 billion [6]. Beef production is complex and involves multiple stages, wherein calves are birthed, raised and fed for slaughter, and processed for meat. The raising of cattle in high-throughput production typically involves the movement of animals from (I) cow-calf systems (a permanent herd used to produce young beef cattle), to (II) backgrounding (post-weaning intermediate feeding, typically forage-based diets), and (III) feedlot/finishing operations (concentrated animal feeding, typically with high-energy grain-based diets). After finishing, animals are transported to a slaughterhouse and processed. Antimicrobials may be given to live cattle at any production stage for therapeutic and non-therapeutic purposes.

## Antimicrobial usage in beef production

### Rationale for antimicrobial use

Antimicrobials are used in beef cattle for the therapeutic treatment of infections caused by bacteria or other microbes. Cattle can be afflicted by a variety of endemic infectious diseases, which may exist ubiquitously in the ranching environment [7]. Endemic pathogens often go unnoticed, but compromise animal health—affecting herd growth performance and farm profitability. Infections spread rapidly in high-density feedlots, and despite herd management procedures, both endemic and exotic diseases can be introduced by importation of diseased animals into the beef production system. Globally, 4.7 million cattle are exported to beef producing countries, with the top exporters being Mexico, Australia, and Canada, exporting >1.3, >1.2, and >1.0 million cattle, respectively. These cattle are sent primarily to the US, which received >2.2 million cattle in 2015 [6]. The risk of disease transmission creates significant economic pressure for antimicrobial usage to prevent infectious bovine diseases.

### Therapeutic and non-therapeutic use of antimicrobials

Antimicrobial use in cattle is unavoidable for the treatment of infections for which vaccines, bacterins, or alternate therapies are not available. A prevalent, controversial practice involves antimicrobials used in non-therapeutic applications. Judicious antimicrobial use typically requires that diseased cattle are treated individually to maximize therapeutic efficacy and reduce the spread of AMR, but entire herds are often dosed with in-feed antimicrobials. This is the typical administration route for practices such as (I) prophylaxis, (II) metaphylaxis, and (III) growth promotion. These practices are described by inconsistent and often agenda-driven terminology. For example, prophylaxis and metaphylaxis are considered therapeutic uses by the American Veterinary Medical Association and the US Food and Drug Administration (FDA) [8, 9], but others consider such practices ‘sub-therapeutic’, ‘non-therapeutic’, or ‘production usage’. More recently, the FDA uses ‘production purposes’ to refer to antimicrobial usage with the intent of growth and feed efficiency enhancement [10]. Prophylaxis is action taken to prevent disease and involves the administration of antimicrobials to an individual that is perceived to be at risk of developing disease. Metaphylaxis refers to the treatment of a larger cohort or entire herd to provide: (I) therapy to infected animals, and (II) prophylaxis to uninfected or potentially susceptible animals. Metaphylaxis is often applied to herds receiving new animals. Growth promotion refers to the use of antimicrobial growth promoters (AGPs) for extended duration to improve feed efficiency (the ratio of feed consumed vs. animal weight gain). ‘Sub-therapeutic’ typically refers to low-dose concentrations of antimicrobials in feeds over an extended duration. The FDA Centre for Veterinary Medicine defines sub-therapeutic as amounts <200 g per ton (US) of feed for 12 wk [11].

### Complexity of production usage of antimicrobials

Although prophylaxis/metaphylaxis may be a more judicious use of antimicrobials than growth promotion, growth promotion is often a benefit of either treatment. For example, antimicrobial treatment and prevention of cattle liver abscesses simultaneously provides prophylactic/metaphylactic therapy and growth promotion. Liver abscesses occur frequently in cattle, and are common in feedlots, where high-energy grain-based diets can cause acidosis, leading to ruminal lesions that predispose cattle to hepatic disease caused by invasive bacteria [12]. Cattle with liver abscesses have reduced production efficiency (reduced feed intake and weight gain) [12]. Thus, feedlot cattle receiving antimicrobials for liver abscess control can also indirectly exhibit growth promotion as a result of disease prevention. Some antimicrobials are approved for both growth promotion and therapeutic applications [13, 14]. Some countries, particularly in the EU, have banned the use of AGPs in beef and other meat production industries (the EU ban was implemented in 2006 [15]). In 2012, the US introduced a voluntary ‘ban’ on AGPs, and a similar program is expected in Canada [16]. While such policies are laudable, their effectiveness is questionable. For example, the volume of agricultural antimicrobials used within the EU has not decreased, and the EU ban may also have resulted in compensatory increases in the usage of antimicrobials with even greater relevance to human health [17]. Regardless, bacterial resistance acquired in response to any antimicrobial usage could compromise future efficacy, especially in the case of AMR genes that are genetically linked in clusters, as is often the case in multi-drug resistant (MDR) organisms.

## Global veterinary antimicrobial usage

Antimicrobial usage data is scarce: most countries do not survey or collect usage data, and cattle producers and pharmaceutical companies have little incentive to report such information. Where usage data exists, typically in high-income countries, it takes the form of volume sales data rather than actual usage. The caveat of antimicrobial sales and distribution data is that it does not accurately indicate how or if antimicrobials were used. In a global analysis of antimicrobial usage, Van Boeckel et al. [18] estimated the worldwide consumption of antimicrobials in food animal production at ≥57,000 t (1 t = 1,000 kg) and projected a 67% increase in total usage by 2030 to ≥95,000 t. Total food-animal antimicrobial sales in the US was reported to be approximately 9,475 t (2014) [19], 8,122 t in the EU (2013) [20], 1,127 t in Canada (2012) [21], 644 t in Australia (2010) [22], and 429 t in the United Kingdom (UK) (2014) [23] (Fig. 1b; excludes ionophores sales). Based on these sales data, and estimations of food animal populations, Van Boeckel et al. projected that the top countries consuming antimicrobials in livestock production are China, the US, India, Brazil and Germany, with China accounting for 23% of global consumption [18].

Data for antimicrobial usage by animal type is not routinely available, such that the proportion and type of antimicrobials sold exclusively for use in cattle is largely unknown or estimated. Some information can be gleaned from country data where specific antimicrobial formulations with indicated routes of administration (e.g. in-feed, injection etc.) are provided for specific livestock (Fig. 1c). However, this data is largely unreliable because (I) most antimicrobials are approved for use in multiple food-animal species, (II) off-label non-intended usage of antimicrobials is a common practice worldwide, and (III) the antimicrobial may not have actually been administered to the animal. Data on therapeutic vs. non-therapeutic use is not collected, and difficult to estimate. Without reliable antimicrobial usage data to link to AMR, it is challenging to create scientific policies to optimize veterinary antimicrobials. Thus, judicious use policies in some countries are the subject of debate, with critics decrying heavy-handed bans and regulations, and proponents criticizing ineffective and optional compliance schemes.

One method to improve antimicrobial usage estimate by species is to take into account (I) the size of the animal population (demographics), and (II) the average theoretical weight of the animal species at time of treatment (physiology). This is the population correction unit (PCU), and is used in the UK Veterinary Medicines Directorate UK-VARSS report [23], the EU European Medicines Agency ESVAC report [20], and the Public Health Agency of Canada’s CIPARS report [21]. Briefly, 1 PCU = 1 kg of livestock, such that the amount of antimicrobials sold can be normalized by species weight, allowing for a comparative indication of overall usage between species (Fig. 1d). Van Boeckel et al. used PCU values to estimate global consumption of antimicrobials per kg of animal produced at 45 mg/PCU (= mg/kg) for cattle, 148 mg/PCU for chickens, and 172 mg/PCU for pigs [18]. This trend is consistent with UK-VARSS data, in which cattle consumed 8 mg / PCU of antimicrobials compared to 172 mg / PCU for swine and poultry [24]. This approach gives an appreciation for the overall use of antimicrobials within a livestock species, but does not indicate usage within the various segments of the production system. These are limitations of using antimicrobial sales and distribution data as a proxy for actual usage data [23].

In some countries, the majority of antimicrobials manufactured or sold are used in food animals rather than in human medicine (e.g. US: ~10,670 t active ingredient for food animals (2014) vs. ~3,290 t for humans (2012) [19, 25]; EU: ~7,982 t active ingredient for food animals vs. ~3,399 t (2012) [26] (food animal values exclude ionophores and other non-medically important antimicrobials)). However, direct human-animal antimicrobial use comparisons are limited by differences in estimation and measurement methodology (e.g. antimicrobials sold vs. prescribed), differences in animal physiology and antimicrobial use practices, and are further complicated by the inclusion/exclusion of antimicrobials irrelevant to human medicine (e.g. ionophores). Thus, food animal vs. human antimicrobial consumption comparisons must be interpreted with caution. Since food animals outnumber/outweigh the human population, volume usage is less surprising than the concurrent use of antimicrobials essential for human medicine. The FDA reports that medically important antimicrobials accounted for 62% of sales of all antimicrobials approved for use in food-producing animals [19], with 74% of clinically relevant antimicrobials administered in-feed [19]. Of the 38% of antimicrobials sold that were not medically important, 80% were ionophores (e.g. monensin). Ionophores are not used in human medicine, have no human counterpart, and do not appear to promote AMR. However, ionophores are important for animal welfare, and are administered for production and therapeutic indications for the treatment/prevention of coccidiosis, a disease associated with *Eimeria* spp*.* infestations [24]. In the EU, ionophores are defined as anticoccidials/coccidiostats, and are not reported as antimicrobials [20, 23]. Besides the ionophores and another class of AGPs called flavophospholipols, most veterinary antimicrobials are identical or structurally similar to antimicrobials used in human medicine. Stringent EU policies regulate the use of in-feed antimicrobials, and penicillins sales are proportionally high-from a low of 11.9% in France to as high as 61.3% in Sweden of all veterinary antimicrobials sold [20]. Sweden was the first country to ban AGPs in 1986 [17], a policy that likely contributed to high therapeutic use of penicillins. Resistance to an agricultural antimicrobial may confer resistance to the human drug, many of which are considered to be essential medicines by the World Health Organization (WHO) [27]. Significant veterinary antimicrobials generally include tetracyclines, penicillin (penam) and other β-lactams, macrolides, sulfonamides, and aminoglycosides (Fig. 1e). Other antimicrobials represent a miniscule fraction of veterinary antimicrobials sold and distributed (each <2%), but they are not unimportant. Thus, cephalosporins, lincosamides, phenicols, and fluoroquinolones (among others) include some of the most effective antimicrobials in veterinary and clinical medicine.

## Antimicrobial resistance in bovine pathogens

Much focus on AMR in food animals concerns the hazards for human health, but AMR is also a veterinary problem. Knowledge about resistance in exclusively bovine pathogens is also exceptionally poor compared to that of bovine zoonotic enteric pathogens, such as *Campylobacter*, *Salmonella*, *E. coli* and *Enterococcus* spp. These species are typically used as ‘indicators’ of AMR in production animals as they (I) are of importance in human disease, (II) are relatively easy to culture, (III) can be isolated from healthy animals, and (IV) have established AMR minimum inhibitory concentration (MIC) breakpoints (for human infections). To reiterate, for several of the bacterial species discussed below, the designation of “resistant” or “sensitive” is often author-determined because clear criteria have not been established by relevant standardization bodies, such as the Clinical Laboratory Standards Institute (CLSI), and the European Committee on Antimicrobial Susceptibility Testing (EUCAST). Surveillance programs monitoring AMR in beef production are typically constrained to human enteropathogens and sentinel AMR indicator species, but independent research from many countries gives rough estimates of AMR in cattle pathogens. Several recent studies have found strong correlations between the level of use of specific antimicrobials and the level of resistance observed [28, 29].

**Fig. 2:**
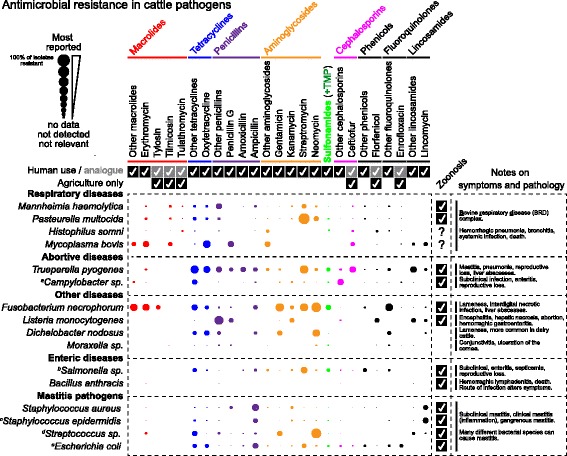
Most frequently reported antimicrobial resistance in pathogens from diseased bovines. Diameter of circle indicates the percent resistance of phenotypic resistance to antimicrobials, by class. The percent resistance was determined via the median of percent values obtained from journal articles (references [30–88]) that reported the percentage of resistance among isolates collected from diseased animals or from passive surveillance (as indicated). Notes: ^*a,b*^includes resistance data from healthy animals; ^*c,d,e*^includes data from healthy animals, sub-clinical, and clinical mastitis; ^*e*^includes isolates from feces. Data compiled from multiple sources

Scientific literature pertaining to AMR in pathogens of significance to beef production was reviewed, and the median percent resistance of 16 different pathogens to antimicrobials was collected from 58 scientific reports ([30–88]; 2000-present), shown in Fig. 2 (see Methods for details). Reports were selected if they contained an antibiogram of isolates without prior antimicrobial selection, and in most cases, if the isolates were obtained from diseased animals. In general, differing levels of tetracycline resistance were present in most cattle-associated bacteria. Macrolide resistance was often reported in BRD pathogens, and in liver abscess pathogens. For almost every species there was a report of resistance to at least one antimicrobial from each major antimicrobial class. A caveat of many of the studies selected is that MIC resistance/sensitivity breakpoint criteria have not been defined for many cattle pathogens, as well as some antimicrobials (e.g. streptomycin). Complicating a general view of resistance across multiple species are the following caveats: (I) some studies do not test the same antimicrobials as others, (II) for some species, reports are very scarce, (III) some studies test relatively few isolates for resistance, (IV) in some cases, designation of resistance is defined by the author and not via standardized interpretive criteria, and (V) the median value of percent of resistance is biased towards values for which there are fewer comparative data points. Thus, the data presented in Fig. 2 should be viewed with caution.

### Antimicrobial resistance in bovine respiratory pathogens

Bovine Respiratory Disease (BRD) is the most frequent and economically important of the primary cattle diseases [89]. Approximately 15% of cattle in North America are treated for BRD, which accounts for ~70% of cattle morbidity, and ~40% of all mortality in feedlots [90]. BRD control is thus a major target of antimicrobial usage [90, 91], and possibly an important source of AMR pathogens. BRD involves a complex of etiological agents including *Mannheimia haemolytica,* the predominant agent [92], *Pasteurella multocida*, and *Histophilus somni* [92, 93]. *H. somni* occurs sporadically, and can cause fatal septicemia in cattle. *Mycoplasma bovis* is also frequently associated with BRD [94]. These ubiquitous pathogens are often described as commensals because colonization is asymptomatic in most healthy animals. As opportunistic pathogens, respiratory disease may develop with detrimental changes to the immune status of the host animal as a result of stress (e.g. transportation, weaning) or viral infections (e.g. Bovine Herpes Virus-1, Bovine Respiratory Syncytial Virus) [89]. Typing of *M. haemolytica* isolates obtained from fatal pneumonia cases in calves show substantial diversity [95], suggesting that outbreaks of BRD are not due to the herd-wide transmission of a single virulent strain, but originate from formerly commensal strains [95, 96]. In North America and many countries, macrolides are often given as BRD metaphylaxis to asymptomatic animals in the presence of diseased animals. Individual cattle symptomatic for BRD may also be treated with a wide range of antimicrobials, with the fluoroquinolone marbofloxacin used in this manner [97]. Clinical symptoms may only become apparent after pulmonary damage has occurred. Consequently, metaphylactic control of BRD often improves the welfare of cattle as well as financial returns through cost savings achieved by reduction in morbidity and mortality [98].

In calves experimentally infected with *M. haemolytica* (4 × 10^7^ CFU), Lhermie et al. [97] demonstrated that low-dose (2 mg/kg) marbofloxacin 12 h after inoculation eliminated this pathogen from all calves, but at 45 h post-inoculation a high-dose (10 mg/kg) failed to do so. Since *M. haemolytica* persisted after this high-dose, a higher risk for AMR development may have been created by a practice thought to be more judicious than mass medication [97]. Thus, although metaphylactic approaches may expose more bacteria to antimicrobial selection, they may also reduce pathology, and eliminate pathogens more effectively than single-dose therapeutic approaches. In another study, continuous sub-therapeutic administration of the macrolide tylosin (Tylan, Elanco; 11 mg/kg in-feed) had no effect in reducing carriage of *M. haemolytica* in beef cattle, compared to substantial reductions after therapy with a single subcutaneous injection of tilmicosin (Micotil, Elanco; 10 mg/kg) or tulathromycin (Draxxin, Pfizer; 2.5 mg/kg) [99]. Antimicrobial usage in single animals has been shown to increase the risk of isolating both susceptible and MDR *M. haemolytica* from pen mates, highlighting the importance of bacterial transmission in the dissemination of AMR [100]. Furthermore, Klima et al. [101] found that MDR occurred more frequently in diseased than healthy cattle (37% vs. 2%) in *M. haemolytica* collected from healthy cattle vs. cattle with clinical BRD. In that study, tetracycline resistance (18%) was the most prevalent resistance phenotype [101]. Resistant *M. haemolytica* and *P. multocida* can also be recovered from diseased antimicrobial non-treated cattle. Via the pan-European VetPath susceptibility monitoring program, de Jong et al. [45] analyzed isolates collected between 2002 and 2006 from diseased cattle with no antimicrobial exposure for at least 15 d prior to sampling, and found that 14.6% of *M. haemolytica* (231 total isolates) were resistant to tetracycline, and 5.7, 3.5 and 0.4% of *P. multocida* (138 total isolates) were resistant to tetracycline, spectinomycin, and florfenicol, respectively [45].

MDR has also been reported in BRD agents. Lubbers et al. [102] evaluated records from 2009 to 2011 from the Kansas State Diagnostic Laboratory for co-resistance in *M. haemolytica* to 6 antimicrobial classes including ceftiofur, danofloxacin and enrofloxacin, florfenicol, oxytetracycline, spectinomycin, tilmicosin and tulathromycin. They found that in 2009, ~5% of isolates were resistant to 5 or more antimicrobials as compared to ~35% in 2011 [102]. *M. haemolytica* isolates resistant to oxytetracycline were 3.5-fold more likely to be resistant to 1 or more antimicrobials, compared to non-oxytetracycline-resistant isolates [102]. MDR has been detected in *P. multocida* and *H. somni*. Klima et al. [92] isolated *M. haemolytica*, *P. multocida* and *H. somni* from BRD mortalities, and determined that 72% of *M. haemolytica* and 50% of *P. multocida* isolates exhibited AMR. Surprisingly, 30% of *M. haemolytica* and 12.5% of *P. multocida* were resistant to >7 antimicrobial classes, including aminoglycosides, penicillins, fluoroquinolones, lincosamides, macrolides, pleuromutilins, and tetracyclines [92]. The MDR isolates originated from feedlots in Texas or Nebraska. MDR was found in multiple *M. haemolytica* populations, suggesting that a clonal population was not responsible for this observation [92]. MDR was due to a tandem array of AMR genes concentrated within an Integrative and Conjugable Element (ICE), a mobile genetic element (MGE) [92]. These elements constitute a diverse group of MGEs found in both Gram-positive and -negative bacteria, and are notable for encoding the conjugation machinery required for mobilisation of ICE to other bacteria, where they often integrate into multi-copy genes such as tRNAs and rRNAs. ICEs also frequently encode virulence factors, heavy metal transporters, and toxin-antitoxin systems, thought to ensure the stability of chromosomally-inserted ICE within cells.

**Fig. 3:**
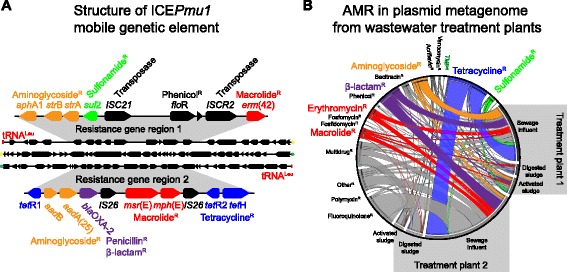
Antimicrobial resistance determinants in mobile genetic elements. **a** Organization of the Integrative and Conjugative Element (ICE) ICE*Pmu1* found in the BRD agent *Pasteurella multocida* [179]. Resistance gene clusters 1 and 2 are shown expanded in grey. **b** Circular distribution of antimicrobial resistance genes by class, and abundance in total annotated antimicrobial genes found six plasmid metagenomes from the influent and sludge from two wastewater treatment plants (modified and reproduced with permission from [192])

A putative ICE, designated ICE*Mh1*, was recently detected in *M. haemolytica* strain 42548 by Eidam et al. that carried resistance to aminoglycosides (*aph*A-1, *str*A, *str*B genes), tetracyclines (*tet*(H) gene), and sulfonamides (*sul*2 gene) [103, 104]. ICE*Mh1* has a size of 92 ,345 bp, harbors ~107 genes, and shares a high degree of similarity with ICE*Pmu1*, an ~82 kb element identified in *P. multocida* that encodes ~88 genes [104]. The structure of ICE*Pmu1* is depicted in Fig. 3a. ICE*Pmu1* integrates into a chromosomal copy of tRNA^Leu^ [105]. Eleven resistance genes are encoded within two gene clusters, conferring resistance to tetracyclines (*tet*R-*tet*(H) genes), streptomycin (*str*A and *str*B), streptomycin/spectinomycin (*aad*A25), gentamicin (*aad*B), kanamycin/neomycin (*aph*A1), phenicols (*flo*R), sulfonamides (*sul*2), macrolides/lincosamides (*erm*(42) gene) or tilmicosin/tulathromycin (*msr*(E)-*mph*(E) genes) [92, 105]. ICE*Pmu1* was shown to conjugatively transfer in vivo into recipient *P. multocida*, *M. haemolytica* and *E. coli* at frequencies of 1.4 × 10^−4^, 1.0 × 10^−5^ and 2.9 × 10^−6^ respectively [105]. *E. coli* transconjugants demonstrated up to 64-fold higher MIC values for florfenicol, suggesting better functional activity of FloR in *E. coli* [105]. A β-lactam oxacillinase (*bla*_OXA-2_) was also present, and conferred greater ampicillin resistance in *E. coli* harboring ICE*Pmu1* [105]. As many of the ICE*Pmu1* resistance genes may not be indigenous to Pasteurellaceae, acquisition of AMR determinants from Enterobacteriaceae is likely [105]. ICE*Pmu1* and ICE*Mh1* were isolated from feedlot BRD cases in Nebraska in 2005 and Pennsylvania in 2007, respectively [104, 105]. There is currently little information on the prevalence of these or similar ICE elements in herds, but the presence of AMR-ICEs in BRD agents represents a critical risk for the efficacy of future antimicrobial therapy. Simultaneous and rapid acquisition of multiple resistance genes via a single HGT event could severely limit therapeutic options.

Besides HGT via MGEs, AMR determinants arise spontaneously via mutation. In some isolates of *M. haemolytica* and *P. multocida*, high-level (MIC ≥ 64 mg/L) macrolide resistance has been attributed to mutations in the multicopy 23S rRNA genes (e.g. *M. haemolytica* A2058G; *P. multocida* A2059G) [106]. Resistance to macrolides, lincosamides and other ribosome-targeting antibiotics has been shown to be conferred by monomethylation of the *M. haemolytica* and *P. multocida* 23S rRNAs at position A2058 [107]. Methylation is catalyzed by a novel monomethyltransferase, designated *erm*(42), which appears to have been disseminated among the Pasterellaceae [107]. Plasmid borne transfer of AMR genes may also be significant among BRD bacteria. In the first report of a *flo*R florfenicol resistance gene in *M. haemolytica*, Katsuda et al. [108] identified pMH1405, a 7.7 kb florfenicol resistance plasmid, which appears to be remarkably similar to plasmids from *P. multocida* (pCCK381; 10.8 kb) and *Dichelobacter nodosus* (pDN1; 5.1 kb). Collectively, these findings reveal the importance and diversity of AMR and HGT mechanisms in BRD pathogens.

### Antimicrobial resistance in liver abscess pathogens

Liver abscesses in beef cattle result from aggressive grain-feeding, and represent an economic liability. Liver abscess incidence in North American feedlot cattle ranges from 12 to 32% [12]. *Fusobacterium necrophorum*, an anaerobic rumen bacterium, is the major etiological agent isolated from condemned livers, followed closely by *Trueperella pyogenes* [12]. Hepatic disease is detected after slaughter since cattle with abscesses are usually asymptomatic. Liver perforation that leads to systemic infection is rare. In-feed antimicrobials, such as the FDA-approved tylosin, chlortetracycline, oxytetracycline, bacitracin, and the streptogramin, virginiamycin, are approved for liver abscess prevention in many countries. In a study of ~7,000 feedlot cattle, tylosin reduced the incidence of liver abscesses by up to 70%, and increased weight gain by 2.3% [12, 109]. Although a common rumen inhabitant, *F. necrophorum* is an opportunistic pathogen also associated with calf diphtheria and foot rot [110]. In a 2-year comparison of flora isolated from liver abscesses in cattle fed with or without tylosin, Nagaraja et al. [111] found that the incidence of *T. pyogenes* in mixed culture with *F. necrophorum* was higher in abscesses from tylosin-fed cattle (53% vs. 10% in the non-tylosin fed cattle). In contrast, the incidence of *F. necrophorum* was higher in cattle that were not fed tylosin (61%), as compared to those that were (33%). No differences in tylosin susceptibility between isolates from antimicrobial-free or tylosin-exposed cattle were identified [111]. AMR in *Fusobacterium* spp. isolated from humans is also relatively rare [112, 113], suggesting that AMR in this genera is yet to present a major risk to beef production or human medicine. AMR in bovine *T. pyogenes* is of greater concern, due to the versatility of the bacterium as a cause of liver, skin, joint, and visceral abscesses, and roles in mastitis and abortion [114]. Tylosin resistance has been documented and linked to the presence of *erm*(X) or an *erm*(B) gene similar to that found on the *Enterococcus faecalis* MDR plasmid pRE25 [115, 116]. This suggests AMR transfer occurs between these human and cattle pathogens. Jost et al. [116] examined 48 *T. pyogenes* isolates, of which 27 were derived from cattle, and identified *erm*(X) as the most prevalent tylosin resistance determinant. An *erm*(X) tylosin and tetracycline *tet*(33) resistance plasmid, pAP2, was also identified [116]. Other studies have found high prevalence of tetracycline and sulfonamide resistance, and suggest that AMR in *T. pyogenes* may of greater significance in bovine mastitis as compared to liver abscesses [117, 118].

### Antimicrobial resistance in keratoconjunctivitis pathogens

Infectious bovine keratoconjunctivitis is a painful ocular disease caused primarily by non-self-limiting infections with *Moraxella bovis* and *bovoculi*. The disease is common worldwide in cattle, transmitted by flies, and if untreated, may result in ulceration and cornea rupture. In the US, only oxytetracycline and tulathromyin are approved for the treatment of bovine keratoconjunctivitis, although penicillin may be used in other countries. In a study of 32 *Moraxella* spp. isolated from cattle and sheep, Maboni et al. [119] found that 40% of isolates were penicillin-resistant and 20% were tetracycline-resistant, but most were susceptible to other antimicrobials. Dickey et al. [120] published the genome sequence for an AMR isolate of *Moraxella bovoculi*, Mb58069. It was found to be resistant to florfenicol, oxytetracycline, sulfonamides, and displayed intermediate resistance to macrolides. Ten AMR determinants were co-located on a >27 kb genomic island [120]. The biofilm-forming capabilities of *Moraxella bovis* may also enhance antimicrobial resistance. Prieto et al. [121] found that *Moraxella bovis* readily forms biofilms, increasing resistance to ampicillin, chloramphenicol, gentamicin, and oxtetracycline by 256-, 1,024-, 512-, and 1,024-fold as compared to when this bacterium grows planktonically [122] Thus, antimicrobial susceptibility via standard disk diffusion and microtiter MIC determinations failed to reflect the true level of resistance of this isolate.

### Antimicrobial resistance in notifiable/reportable bovine bacterial pathogens

Many countries maintain registries of notifiable diseases associated with zoonotic, unvaccinable, highly infectious, economy-damaging, or largely untreatable pathogens. For cattle, notifiable diseases include (I) abortive agents: *Brucella abortus* (Brucellosis), *Coxiella burnetti* (Q fever), and *Leptospira* spp. (Leptospirosus); (II) bovine pneumonia agents: *Mycoplasma mycoides* subsp. *mycoides* small colony type (Contagious bovine pleuropneumonia), and *Mycobacterium bovis* (Bovine tuberculosis); and (III) enteritis agents: *Mycobacterium avium* subsp. *paratuberculosis* (Johne’s disease), and *Bacillus anthracis* (Anthrax) [123]. Although it might be assumed that AMR would be a major issue in these pathogens, for the most part AMR has not been studied in these pathogens or is rare. Besides the rarity of cases, other reasons for this include: (I) the notifiable pathogen is already intrinsically resistant to many antimicrobials (e.g. *Mycobacterium* spp.); (II) the pathogen resides in an antimicrobial-exclusive intracellular niche that renders antimicrobial therapy impractical (e.g. *Brucella abortus* and *Coxiella burnetti*); or (III) a secreted toxin causes pathology (e.g. *Bacillus anthracis*). Control of outbreaks of these diseases rarely involves antimicrobial therapy and relies on animal segregation, herd control, or depopulation [13].

AMR susceptibility tests of human clinical isolates of *Mycobacterium bovis* have been performed because of the role of *M. bovis* in human tuberculosis (TB). Although it can infect many species, the main reservoir of *M. bovis* is cattle, and transmission to humans is primarily via contact with infected animals and drinking unpasteurized milk [124]. In clinical isolates of *M. tuberculosis* and *M. bovis* collected over 15 yr, Bobadilla-del Valle et al. [125] found that 16.6% of isolates from human TB cases were *M. bovis*. Susceptibility testing to first-line anti-TB drugs revealed that 10.9% of *M. bovis* were streptomycin-resistant, and 7.6% were MDR (isoniazid- and rifampin-resistant). The aminoglycoside streptomycin is approved for use in cattle against aerobic Gram-negatives such as enteritis-causing *E. coli* and *Salmonella* spp. [14]. Bovine-human transmission of AMR *M. bovis* appears to be rare in developed countries, but may occur more frequently in developing countries [124, 126].

## Antmicrobial resistance in zoonotic human enteropathogens

### Antimicrobial resistance in bovine-origin *Escherichia coli*

Cattle are *E. coli* reservoirs, with most strains harmless commensals. Some *E. coli*, particularly invasive and enterohemorrhagic *E. coli* (EHEC) cause septicemia in neonatal calves, but are primarily pathogenic to humans. *E. coli* strains from bovines and other food production animals serve as indicators of AMR prevalence in Gram-negative bacterial populations, thus sentinel ‘generic’ *E. coli* help establish and track the persistence of AMR genes in environments affected by beef production and other human activities. For example, in a recent survey of AMR in *E. coli* from Nebraska cattle feedlot runoff catchment ponds and the effluent of municipal wastewater treatment plants, Agga et al. [127] found that the diversity of AMR genes in human-associated samples was greater than from environments impacted by cattle. Interestingly, *E. coli* resistant to 3^rd^ generation cephalosporins and trimethoprim/sulfamethoxazole were found at equivalent high-frequency (>70% of *E. coli* isolates) in both livestock and municipal wastewater environments [127].

Extended-spectrum β-lactamases (ESBLs) that inactivate newer cephalosporins are a major focus of sentinel *E. coli* susceptibility testing. Cottell et al. [128] evaluated *E. coli* originating from 88 steers that were treated with ceftiofur and/or chlortetracycline in an experimental US feedlot. The ESBL *bla*_CTX-M-32_, was detected in cefoxatime-resistant *E. coli* in 29 animals, and was found to be present on a self-transmissible IncN-family plasmid (reviewed in [129]). In Germany, *bla*_CTX-M-1_ was the predominant ESBL in *E. coli*, found on 87% of assessed farms [130]. In a Swiss study of the wider food processing chain, Geser et al. [131] screened for ESBL in fecal samples collected at slaughter as well as in raw milk, and minced beef. They found that of 124 bovine fecal samples 13.7% hosted ESBL-producing bacteria, 98% of which were *E. coli*. Despite enrichment for ESBL-producing organisms, ESBL were not detected in raw milk or minced beef samples. The ESBLs detected in the study included *bla*_CTX-M-1_, *bla*_TEM-1_ *bla*_CTX-M-14_, *bla*_CTX-M-117_, and *bla*_CTX-M-15_. Many of the ESBL-positive isolates were frequently co-resistant to tetracycline (76%), trimethoprim/sulfamethoxazole (76%), nalidixic acid (47%), at least one aminoglycoside (76%), chloramphenicol (65%) and ciprofloxacin (41%). The authors suggested that slaughter hygiene prevented the transmission of ESBLs into the food chain [131]. Similarly, the prevalence of AMR *E. coli* O157:H7 was investigated in 510 fecal, hide, carcass, and raw meat samples from 4 beef slaughterhouses in China. STEC was detected in 1.4% of fecal and hide sample, but not in pre- and post-evisceration carcasses, nor in raw meat samples, with all isolates sensitive to 16 relevant antimicrobials [132]. During slaughter, cattle hides are major contributors to carcass contamination [133, 134]. In another study tracking *E. coli* resistant to 3^rd^-generation cephalosporins or trimethoprim/sulfamethoxazole, Schmidt et al. [135] determined the prevalence of generic and AMR *E. coli* at various sites along the beef processing continuum. The prevalence of cephalosporin-resistant and trimethoprim/sulfamethoxazole-resistant *E. coli* in fecal samples at processing was 75 and 95%, respectively. Prevalence in pre-evisceration carcasses was 3 and 33%, and resistant isolates were only found in 0.5% of final carcasses, and no isolates were associated with the final striploin product. All cephalosporin-resistant *E. coli* isolated were resistant to ampicillin, ceftiofur, and ceftriaxone, and 64% of isolates harbored *bla*_CMY_, conferring additional resistance to clavulanate/amoxicillin and cefoxitin [135]. These reports suggest that hygienic practices in beef processing are effective against AMR bacteria.

### Antimicrobial resistance in bovine-origin *Salmonella*

Non-typhoidal *Salmonella* spp. (often *Salmonella enterica* serotype Typhimurium or Enteritidis) are frequent laboratory-confirmed infectious agents of gastroenteritis. Although the enteritis is usually self-limiting, invasive *S. enterica* spp. infections often require antimicrobial therapy. Cattle are infected/colonized by many *Salmonella* species, and ground beef is a vehicle of *Salmonell*a transmission, implicated in 45% of outbreaks linked to beef [136]. In cattle, susceptible adults develop enteritis, and calves may also develop septicemia. *S. enterica* serotypes Dublin and Newport are associated with bovine salmonellosis, and adult cattle may carry and shed *Salmonella* asymptomatically for many years. In humans, serotype Dublin has the highest proportion of invasive infections resulting in hospitalization and mortality [137]. Due to the frequency of infections, the development of AMR in *Salmonella* is a risk to human health. In North America, MDR *Salmonella* are on average resistant to 7 antimicrobials [138]. In the US, *Salmonella* (and other enteropathogens) are collected from humans, animals, and retail meat for the National Antimicrobial Resistance Monitoring System (NARMS) [137]. In 2013, *Salmonella* was isolated from 7.9% of beef cattle, and in 0.9% of ground beef samples [137]. MDR (>3 antimicrobials) was found in 20% of all ground beef serotype Dublin isolates, many of which were resistant to ampicillin, chloramphenicol, streptomycin, sulfonamides, and tetracycline [137]. Worse still, the prevalence of ceftriaxone resistance (3^rd^ generation cephalosporin) in bovine-origin serotype Dublin increased from 0 to 86% between 1996 and 2013 [137]. As this is a major risk to human health, adoption and adherence to good practices during beef processing and proper cooking are critical to prevent transmission [136, 139, 140].

### Antimicrobial resistance in bovine-origin *Campylobacter*

*Campylobacter* is the most frequent cause of human bacterial gastroenteritis in the developed world, with *Campylobacter jejuni* responsible for >90% of *Campylobacter* infections [141]. Mostly a self-limiting infection in humans, severe cases of campylobacteriosis are treated with drugs such as erythromycin or ciprofloxacin. *Campylobacter* are frequent colonizers of chickens, but cattle are an important reservoir, and can carry high numbers of *Campylobacter* asymptomatically [142]. Susceptible cattle can suffer from enteritis, and *Campylobacter fetus* subsp. *fetus* and subsp. *venerealis* can cause venereal bovine genital campylobacteriosis, leading to infertility and abortion [13, 142]. In the NARMS report, *Campylobacter* was isolated from 42% of beef cattle, with 14% of isolates resistant to ciprofloxacin [137]. In a Japanese study of beef cattle, *C. jejuni* was isolated from 36% of cattle on 88% of the farms surveyed: ~40% of *C. jejuni* isolates were enrofloxacin- and nalidixic acid-resistant, but none were erythromycin-resistant [143]. In a Swiss study of 97 *Campylobacter* isolates obtained from a beef processing plant, Jonas et al. [144] found that 31% were fluoroquinolone-resistant and ~1% were erythromycin-resistant. Wieczorek et al. [145] examined *Campylobacter* abattoir prevalence on 812 bovine hides and corresponding carcasses, and found *Campylobacter* on 25.6% of hides, and 2.7% of carcasses. The isolates obtained were equally resistant to nalidixic acid and ciprofloxacin (38.3%), streptomycin (24.3%), tetracycline (20.9%), erythromycin (4.3%), and gentamicin (2.6%) [145].

### Antimicrobial resistance in bovine-origin *Enterococcus*

*Enterococcus* spp. are ubiquitous Firmicutes in the healthy intestinal microbiota of both humans and cattle, and indicate fecal contamination. Most *Enterococcus* spp. are not foodborne pathogens, nor are they bovine pathogens [13]. Despite this, isolates of *Enterococcus faecalis* and *faecium* may cause life-threatening human infections, such as UTIs and meningitis. Control of enterococci infections is complicated by high-level MDR [146]. Enterococci are referred to as ‘drug-resistance gene traffickers’ due to their omnipresence, robustness, and capability of transferring AMR to other species and pathogens [147, 148]. *E. faecalis* transferred gentamicin resistance plasmids to transplanted human flora in a BALB/c mouse model [149]. The US NARMS report indicates that *Enterococcus* were recovered from ~90% of cattle, and ~80% of retail ground beef tested. The incidence of MDR (>3 antimicrobials) in both *E. faecium* and *faecalis* was lower in cecal isolates from beef cattle (19 and 14%, respectively) than in cecal samples from chickens (67 and 46%, respectively) or turkeys (25 and 58%, respectively) [137]. Other studies of AMR *Enterococcus* typically focus on the emergence of resistance to vancomycin— an antimicrobial used in the treatment of MRSA and other Gram-positive infections [122, 150]. Vancomycin or linelozid resistance was not detected in bovine-origin *Enterococcus* spp*.* in the United States or Canada [137, 151], but ~30% of *E. faecium* NARMS isolates were found to be quinupristin/dalfopristin-resistant [137]. Overall, despite the possibility for transmission of pathogenic strains to humans, *Enterococcus* spp. in the beef production environment have been studied mainly for their presumptive importance as AMR determinant sentinels/reservoirs.

## Antimicrobials and the bovine microbiota

Cattle house a dense (>10^10^ microbes/ml; rumen fluid [152]) consortia of microbial species in the distinct physiological niches of the rumen and lower digestive tract [153]. Different host compartments functionally select for, and are shaped by, distinct microbial communities that are essential for the proper physiology and development of the host [154, 155]. Cattle are dependent on rumen microbes for feed digestion, and the microbiome collectively degrades complex polysaccharides, converting plant mass into volatile fatty acids for absorption by the host animal. Core microbial species in the rumen include *Prevotella*, *Butyrivibrio*, *Ruminococcus*, as well as many unclassified organisms [156, 157]. Other bovine niches harbor unique microbial communities, such as the nasopharyngeal and vaginal tracts [153, 158, 159]. The microbial community in the jejunum also has a role in feed digestion, and influences feed efficiency [160]. The fecal microbiota is dominated by *Firmicutes* and *Bacteroidetes*, but also contains *Proteobacteria* and human enteropathogens, which are shed in feces [154, 161, 162]. Collectively, the intestinal microbiota hosts a portion of the cattle resistome.

Unlike in humans and experimental animal models, there is currently limited information concerning the effect of antimicrobials on the bovine microbiota/resistome. However, much work describes the effect of therapeutic and sub-therapeutic administration of antimicrobials on the prevalence of specific bacteria in bovines. These studies typically involve antimicrobial administration to a controlled animal cohort, followed by culture-dependent collection of an organism-of-interest for susceptibility testing. These approaches provide a biased snapshot of microbiome changes. Newer methods include culture-independent collection of metagenomic DNA for detection and quantitation of specific AMR genes by PCR-based methodology, or for high-throughput sequencing and functional AMR gene annotation (Table 1). There are currently few studies describing the effects of antimicrobials on microbial population diversity in bovines using high-resolution sequencing methodology.

**Table 1:** Selected studies on the effect of antimicrobials on the cattle microbial resistome

Study	Livestock (animals in study)	Antibiotic tested (class)	Experimental treatment	Sample type	Characterization methodology	Outcome or notable findings
Chambers et al. 2015 [165]	Dairy cattle (6 Holstein cows)	Ceftiofur (3^rd^ generation cephalosporin)	Administration of therapeutic ceftiofur over 3 d trial	Fecal	Metagenomic DNA: Illumina HiSeq of total DNA with MG-RAST and ARDB annotation	Increase in bacterial sequences associated with resistance to β-lactam and multidrug resistance
Benedict et al. 2015 [175]	Beef cattle (>10,000 animals)	Various (5 difference antimicrobial drug classes	Correlation between routine antimicrobial usage in a feedlot system and antimicrobial resistance in non-type *Escherichia coli* over 3 yr	Fecal	Bacterial isolation and susceptibility testing.	Exposures to tetracycline, streptomycin, and trimethoprim-sulfamethoxazole were significantly associated with increased abundance of antimicrobial resistance genes
Kanwar et al. 2014 [164]	Beef cattle (176 steers)	Ceftiofur (3^rd^ generation cephalosporin) Chlortetracycline (tetracycline)	Administration of therapeutic ceftiofur and/or chlortetracycline over 26 d trial	Fecal	Metagenomic DNA: qPCR of select AMR genes	Increase in ceftiofur resistance genes and decrease in tetracycline resistance genes following ceftiofur treatment Increase in ceftiofur and tetracycline resistance genes following chlortetracycline treatment
Zaheer et al. 2013 [99]	Beef cattle (40 steers)	Tylosin Tulathromycin Tilmicosin (macrolide)	Administration of either sub-therapeutic tylosin or therapeutic tulathromycin or tilmicosin	Fecal	Bacterial isolation and susceptibility testing. PCR of select AMR genes	Both sub-therapeutic and therapeutic macrolide treatment increased abundance of macrolide resistant Enterococci
Thames et al. 2012 [219]	Dairy cattle (41 calves)	Neomycin (aminoglycoside) Oxytetracycline (tetracycline)	Administration of either sub-therapeutic or therapeutic neomycin or oxytetracycline over 50 d milk-replacement trial	Fecal	Metagenomic DNA; qPCR of select AMR genes	Sub-therapeutic antibiotic treatment had no effect on abundance of tested resistance determinants. Therapeutic treatment with oxytetracycline increased abundance of tetracycline resistance genes

### Effect of antimicrobials on the bovine microbiota

Pereira et al. [163] characterized the gut microbiota (fecal samples) of pre-weaned dairy calves fed raw milk spiked with ‘residual’ concentrations of ceftiofur (ceftiofur sodium; 0.1 μg/mL), ampicillin (ampicillin sodium; 0.01 μg/mL), penicillin (penicillin G sodium; 0.005 μg/mL), and oxytetracycline (oxytetracycline hydrochloride; 0.3 μg/mL) using 16S rRNA Illumina MiSeq-based sequencing. Exposure resulted in Genus-level differences, but taxa above the Family level were not altered [163]. The microbiota of exposed calves was also less diverse than treatment-free calves [163]. Similarly, Reti et al. [162] examined the effects of a sub-therapeutic AGP on the abundance and composition of microflora in the small and large intestine of adult beef cattle. The US- and Canada-approved chlortetracycline/sulfmethazine AGP (Aureo S-700 G, Alpharma) was administered at 350 mg of each antimicrobial per head per day for 28 d [14]. Compared to non-treated control cattle, beef cattle administered the AGP showed no differences in bacterial abundance or richness/diversity composition (determined via quantitative PCR and terminal restriction fragment length polymorphism analyses) [162]. Studies using advanced 16S rRNA metagenomic sequence-based and whole metagenome methodologies may be of greater significance in future work exploring the effect of antimicrobials on the microbiota.

### Effect of therapeutic and sub-therapeutic antimicrobial usage on AMR gene prevalence

Kanwar et al. [164] recently explored the effects of differential treatment strategies on the prevalence of AMR determinants in the fecal metagenome. In a 26-day field trial, 176 beef steers were divided into 4 cohorts and given therapeutic doses of ceftiofur (ceftiofur crystalline-free acid (CCFA), Excede, Zoetis; 6.6 mg/kg body weight) and/or chlortetracycline (Aureomycin, Alpharma; 22 mg/kg body weight). One of the four cohorts included steers in which only 1 of the animals was administered ceftiofur and chlortetracycline, while the remaining animals received chlortetracycline alone. Via quantitative PCR, the authors determined gene copies/g of wet feces of *bla*_CMY-2_ and *bla*_CTX-M_ (ceftiofur resistance), *tet*(A) and *tet*(B) (tetracycline resistance), and 16S rRNA genes in fecal community DNA from the pens of each treated cohort. Pens where all cattle were treated with ceftiofur had greater numbers of *bla*_CMY-2_ and *bla*_CTX-M_ ceftiofur resistance determinants than single-animal treatment pens [164]. Chlortetracycline treatment increased the levels of *bla*_CMY-2_ and *bla*_CTX-M_ gene copies compared to cattle in pens that did not receive chlortetracycline. In contrast, tetracycline AMR gene prevalence decreased in pens where all cattle received ceftiofur compared to pens where only one animal received ceftiofur [164]. The authors discussed these findings in the context of expansion or suppression of singly- or co-resistant AMR populations under antimicrobial selection, which served to highlight the complexity of the effects of antimicrobials on the resistome, and the potential for discrepancies between culture- and non-culture-based AMR quantitation methodologies [164].

Utilizing advanced total community metagenomic sequencing, Chambers et al. [165] examined the effect of ceftiofur treatment on the prevalence of AMR genes in the bovine fecal microbiome. Holstein cows were injected subcutaneously with ceftiofur (CCFA, Excede, Zoetis; 1 mg per 45.4 kg body weight) and fecal samples were collected prior to and post-treatment. Total DNA was sequenced on the Illumina HiSeq platform, and AMR genes were detected using the antibiotic resistance genes database (ARDB) [166]. The proportion of β-lactam and MDR sequences were found to be higher in ceftiofur-treated cows relative to control cows. The β-lactamase genes *cfx*A2 and *cfx*A3 were most abundant, and have previously been associated with *Prevotella*—a common rumen microbe [167]. Ceftiofur also changed the fecal bacterial community composition, increasing Bacteroidia and decreasing Actinobacteria. This study was also notable because metagenomic data was functionally assessed with MG-RAST [168], allowing examination of antimicrobial-induced changes to the metagenome. Functional ceftiofur-associated shifts included increased prevalence of genes associated with stress, chemotaxis, and resistance to toxic compounds [165]. This work and others like it likely represent the future direction of AMR surveillance research.

Sub-therapeutic antimicrobial administration is one of the most controversial beef production practices with many studies exploring this topic in the context of AMR development. Alexander et al. [169] investigated effects of chlortetracycline/sulfamethezine AGPs (Aureu S-700 G, Alpharma; 44 mg/kg each in-feed) on the prevalence of AMR *E. coli* in the beef production continuum. With respect to treated and non-treated cattle, *E. coli* was collected from live-animal feces, hides, intestinal digesta, carcasses, and ground beef. Animals fed chlortetracycline/sulfamethezine harbored more tetracycline-resistant *E. coli* than non-treated animals (50.9% vs. 12.6%), but there were no differences in the prevalence or profile of AMR *E. coli* between treatments in the hide, carcass or ground beef samples [169]. To the authors this suggested that AMR *E. coli* can enter the food chain at slaughter regardless of AGP administration [169]. Sub-therapeutic administration of tetracycline/sulfamethazine also increased the prevalence of tetracycline-resistant organisms, and increased the frequency of ampicillin-resistant *E. coli,* in agreement with similar studies using the same antimicrobials [170]. Another study found that sub-therapeutic tylosin treatment (Tylan, Elanco; 11 mg/kg in-feed) increased the frequency of *Enterococcus* spp. harboring *erm*(B) and/or *msrC* (a macrolide/streptogramin efflux pump gene) [171]. The authors of that study concluded that the diversity of *Enterococcus* decreased in the period between when cattle entered and exited the feedlot, and that the AMR *Enteroccocus* were derived from strains present in the intestinal microbiota before tylosin administration [171]. Selection for co-resistance and MDR is one of the main arguments against AGPs.

### Effect of BRD-related antimicrobial usage

Given the importance of antimicrobials in the treatment of BRD agents, much research examines the effect of antimicrobial treatment on AMR development in BRD bacteria. Investigated the effects of therapeutic and sub-therapeutic macrolide administration on the nasopharyngeal and enteric microbiota, with specific focus on *M. haemolytica* and *Enterococcus*, respectively. Forty beef steers were injected once with tilmicosin (Micotil, Elanco; 10 mg/kg) or tulathromycin (Draxxin, Pfizer; 2.5 mg/kg) or fed sub-therapeutic tylosin (Tylan, Elanco; 11 mg/kg in-feed) continuously over 28 d. Therapeutic tilmicosin and tulathromycin decreased nasopharyngeal carriage of *M. haemolytica*: at the beginning of the trial, 60% of the steers tested positive for *M.* haemolytica, at 7 d post- injection, none of the steers treated with tilmicosin harbored *M. haemolytica*, and only one steer treated with tulathromycin was positive for *M. haemolytica*. Sub-therapeutic tylosin had no effect on nasopharyngeal carriage, and tylosin-exposed *M. haemolytica* isolates did not acquire macrolide resistance. In contrast, a significant proportion of the bystander *Enterococcus* acquired *erm*(B) erythromycin resistance following treatment with either injectable tilmicosin or tulathromycin, or in-feed tylosin, and were 76-fold more likely to be erythromycin-resistant than those recovered from non-antimicrobial-treated steers. Catry et al. [172] correlated 2-year of Belgian farm-standard antimicrobial usage to the occurrence of AMR in rectum and nasal flora, represented by *E. coli* and *Pasteurellaceae*, respectively. Narrow spectrum penicillins were the most frequently administered parenteral antimicrobials, often in combination with an aminoglycoside, such as neomycin or dihydrostreptomycin [172]. Among rectal *E. coli*, 20.6% were resistant to least one antimicrobial. The most frequent resistance patterns were ampicillin-tetracycline-streptomycin (15.9%), tetracycline-streptomycin (11.4%), and ampicillin-streptomycin (9.8%) [172]. Among 206 *P. multocida* isolates and 42 *M. haemolytica* isolates originating from the nasal cavity, the predominant resistance found was to the aminoglycoside spectinomycin [172]. The authors confirmed that antimicrobials altered the prevalence of AMR in the digestive and respiratory tracts and highlighted that the route of administration affected resistance outcomes. Individual therapy was linked to increased but transient resistance, whereas in-feed antimicrobials were linked to higher levels of MDR [172]. Others have also suggested that the route of administration affects overall AMR prevalence [173, 174], but there are also contradictory reports where no such association exists [99, 175].

### Heavy metal supplementation and AMR

Cattle also receive trace mineral supplements that include elements with AGP activity. Some heavy metals, such as zinc, manganese, and copper may be given as salt-mixes, injected, or administered in slow-release ruminal capsules [14]. Copper and zinc promote growth, potentially via suppression of pathogens and alteration of microbiota [176, 177]. In other production animals, zinc and copper can select for AMR [178]. This may be due in part to MGEs such as ICE, in which AMR determinants are co-localized with heavy-metal resistance genes. For example, in addition to multiple AMR determinants, ICE*Pmu1* (Fig. 3a) encodes for a multi-copper oxidase, which is potentially involved in resistance to copper and other heavy metals [179]. Thus, heavy metal exposure can co-select for AMR. Jacob et al. [180] studied the effect of elevated copper and zinc fed to heifers receiving high-energy rations by isolating and characterizing AMR *E. coli* and *Enterococcus* from fecal samples. Resistance to copper and zinc in *E. coli* isolates was increased, and abundance of the tetracycline resistance determinant *tet*(M) was elevated following heavy metal supplementation [180]. In a study combining tylosin (Tylan, Elanco; 0 or 10 mg/kg in-feed) with copper (CuSO_4_; 10 or 100 mg/kg in-feed), Amachawadi et al. [181] investigated fecal *Enterococcus* spp. to determine if elevated copper supplementation co-selects for macrolide resistance. The transferable copper resistance gene *tcr*B was identified in 8.5% of *Enterococcus* from elevated copper- and tylosin-fed cattle, compared to copper alone (4.5%), tylosin alone (3.5%), or the low copper/no tylosin control (2.0%) [181, 182]. All the *tcr*B-positive isolates proved to be *E. faecium*, and interestingly, all *tcr*B-positive isolates harbored tetracycline *tet*(M) and erythromycin resistance *erm*(B) determinants [181]. The authors concluded that elevated dietary copper could co-select for AMR in feedlot cattle [181]. Thus, heavy metal supplementation should also be considered as a selective pressure with the potential to promote the dissemination AMR determinants, and is a practice that likely needs to be revisited as these minerals may be added to the diet in excess of the animal’s requirement.

## The bovine resistome & the wider environment

The primary concern relating to antimicrobials in agriculture is the potential for AMR determinants to expand and spread via the food chain. Although urban lifestyles rarely bring people into direct contact with livestock, the animal production continuum extensively connects with numerous industries, infrastructure, and ecologies. For example, manure from antimicrobial-treated animals may be applied to crops, or waste from farms may drain into rivers, reservoirs, and wastewater treatment plants. In the US, cattle produce between 0.86 and 6.4 million t of manure daily [183]. AMR can thus be transferred to the wider environment, increasing the risk of contact with a human pathogen. At present, knowledge about the identity, diversity, distribution, and patterns of co-resistance in beef-related AMR genes, and how they compare to determinants in other ecosystems is scarce, due in part to the difficulty in defining the bovine resistome in the context of the larger environmental resistome. AMR genes are widely present in both pristine and human-impacted environments [184], so the occurrence of AMR in any specific biome does not necessarily validate the impact of antimicrobial usage. However, with the advent of next-generation sequencing and total metagenomics, and resources like ARDB, and CARD (the Comprehensive Antibiotic Resistance Database; [185]), high-throughput AMR gene profiling resistomics is shedding light on these relationships.

### Resistome characterization via shotgun metagenomics

Noyes et al. [186] examined AMR genes of 1,741 beef cattle as they moved longitudinally through the production chain, characterizing feedlot, slaughter, and beef product resistomes via shotgun metagenomics performed on the Illumina HiSeq platform, and assessed against the Resfinder [187], ARG-ANNOT [188], and CARD [185] AMR gene databases. This identified 300 unique AMR genes, and showed that, the diversity of the AMR genes decreased while cattle were in the feedlot, indicative of selective pressure imposed by antimicrobials, consistent with other studies showing diversity reduction following antimicrobial exposure [163]. Examination of post-slaughter samples obtained from belts and tables in the slaughterhouse, meat trimmings, and market-ready samples revealed no AMR genes [186]. The authors concluded that effective practices at slaughter minimized the likelihood of AMR gene being passed through the food chain. However, the high prevalence of bovine DNA complicates shotgun metagenomics and may result in low sensitivity of AMR gene detection. Despite this, this study exemplifies the powerful utility of metagenomic approaches in the study of AMR gene ecology.

Metagenomics have also proved useful in the examination of AMR genes found in wastewater treatment plants associated with tanneries and slaughterhouses. Wastewater treatment plants are thought to be HGT hotspots because of high bacterial diversity and density [189, 190]. Wang et al. [191] profiled AMR genes and MGEs in wastewater sludge from a Chinese leather tannery via Illumina HiSeq and assessment with MG-RAST [168] and ARDB [166]. *Proteobacteria* were most-prevalent in anaerobic and aerobic sludge accounting for 35.95 and 58.36% of annotated reads, respectively, followed by *Firmicutes* (16.31 and 6.08%, respectively) [191]. Concerning AMR genes 747 reads (0.0081%) and 877 reads (0.0101%) in anaerobic and aerobic sludge, respectively, were assigned to 54 and 42 types of known AMR genes [191]. MDR efflux transporters were most common, followed by tetracycline and sulfonamide resistance genes (>20% of AMR-associated reads) [191]. The authors also detected MGEs in tannery DNA samples, but limitations in methodology restricted investigating linkages with AMR genes. Taking a similar approach, Li et al. [192] examined the resistome of plasmids harvested from influent, activated sludge, and digested sludge of two Hong Kong wastewater treatment plants receiving domestic and slaughterhouse (cattle and other production animals) sewage. AMR genes were detected in all of the plasmid metagenomes: the most abundant were tetracycline resistance genes (29% of all AMR gene sequences), quinolone resistance genes (17%), and β-lactam resistance genes (12%) [192]. The AMR gene distribution and abundance in each wastewater treatment plant sample is shown Fig. 3b, in circular relationship format [192, 193]. This plasmid-centric study highlights the mobile resistome and plasmid fates more so than a total metagenome study, and future experiments could involve comparisons between plasmid and total resistomes to explore HGT of AMR determinants. This paper also highlights a methodology to examine MGE-associated AMR genes that is not confounded by environmental AMR genes or host DNA contamination.

### Resistome characterization via functional metagenomic library screening

Sequence-based metagenomic AMR gene profiling is also limited to those genes with similarity to already known AMR genes, and metagenomic shotgun read lengths present difficulties for the characterization of the AMR genomic context. Functional metagenomic library-based approaches have proved to be complementary in the identification, quantification, and characterization of novel resistance determinants. Wichmann et al. [194] examined the resistome of dairy cow manure with large-insert (>35 kb) fosmid libraries constructed from 5 manure samples. The resulting *E. coli*-based libraries (containing 25.9 Gb of DNA) were screened for resistance to kanamycin, chloramphenicol, tetracycline, and the β-lactams carbenicillin (penicillin) and ceftazidime (cephalosporin). Of 87 AMR *E. coli* clones with genes conferring resistance to at least one of the antimicrobials tested, 80 carried unique AMR genes, suggesting that the cow microbiome harbors AMR genes that are unique or unidentified elsewhere. A novel clade of chloramphenicol acetyltransferases was also described [194]. Flanking sequence analysis indicated that the AMR determinants originated from typical cattle microbes: *Firmicutes* were predominant (50% of sequenced clones), followed by *Bacteroidetes* (23%) and *Proteobacteria* (14%) [194]. Another powerful advantage of the fosmid library approach is the ability to examine AMR gene context: i.e. co-occurrence with other AMR genes, or association with MGEs. Wichmann et al. found 2 kanamycin-resistant *E. coli* clones with >5 putative genes with predicted AMR or MGE functions [194]. Thus, library-based functional metagenomic approaches combined with next-generation sequencing are a powerful way to screen for AMR determinants associated with MGEs, plasmids, or phages [195].

## Linking antimicrobial use in beef production to human health risk

### Assessing the differential risk, importance, and source of AMR genes

Given the ubiquity of AMR determinants in bovine and other microbial communities, it is difficult to appraise the relative risk any particular determinant presents for the likelihood of transfer into a human pathogen and clinical therapy failure. Confounding the issue are AMR determinants that are expressed or silent in different hosts, as well as AMR determinants akin to housekeeping genes [196]. For the latter, ‘decontextualized’ housekeeping genes, such as those harbored on MGEs, pose a greater risk [1, 197]. Prioritizing the differential human health risk posed by an AMR gene is complicated by such issues, but risk ranking schemes have been discussed [1, 198, 199]. Greatest risk may be presented by AMR genes already hosted on MGEs in human pathogens, and known to cause therapy failure. An example of this is the recently detected plasmid-mediated colistin (polymyxin E) resistance gene (*mcr-1*) in *E. coli* isolates from poultry, swine, and infected humans [200, 201]. A beef-related example is the ~38 kb R plasmid found in *S. enterica* serotype Newport, which confers resistance to tetracycline, ampicillin, and carbenicillin [202]. This caused severe penicillin-unresponsive salmonellosis linked to contaminated hamburger meat [202]. The next level of risk may be from functional AMR genes conferring resistance to human antimicrobials, but which are hosted in MGEs in non-pathogenic bacteria. These might include the AMR determinants encoded by ICE*Pmu1* and ICE*Mh1* found in *P. multocida* and *M. haemolytica*, respectively [103, 104]. Elevated risk is credited to MGEs because the acquisition and selection of an AMR determinant in a MGE might be the initial step for transmission to a human pathogen. In the future, more focus should be devoted to AMR in the context of MGEs, particularly for total resistome studies utilizing libraries and shotgun metagenomics, or emerging long-read sequencing technologies.

An example of risk and source determination may be given by the long-term global epidemics of ground beef-associated MDR *S. enterica* serotype Typhimurium phage type DT104, which may express resistance to ampicillin, chloramphenicol, streptomycin, sulfamethoxazole, and tetracycline (resistance-type ACSSuT) [203–205]. In some isolates, these AMR genes are hosted in a 13 kb MDR region, residing in a larger chromosome-encoded ~43 kb region called *Salmonella* genomic island 1 (SGI1). The MDR region harbors Class I integrons—genetic elements capable of consolidating multiple AMR gene cassettes [206]. Integrons are often found in conjunction with MGEs; in the case of DT104, HGT can occur via phage-mediated transfer [207]. Although veterinary antimicrobial usage and food animals have long been the chief culprit for the origin and dissemination of DT104, Mather et al. [208, 209] challenged the perception that DT104 originated from a single zoonotic population by whole-genome sequencing Scottish DT104 collections. In total, 135 isolates from humans and 83 from cattle were sequenced and compared against 111 other DT104 isolates from diverse host animals and countries. Using phylogenetic diffusion models, the authors found that AMR DT104 populations were distinguishable between cattle and humans, and that animal-to-human and human-to-animal transitions were rare, and occurred at the same frequency [209]. This suggested that most human infections were unlikely to originate from the local cattle. AMR diversity was greater in human isolates, resulting from multiple, independent recombination events in SGI1’s MDR region [209]. In part, this suggested that most human infections were acquired from humans, and that DT104 circulated separately in the animal and human populations, and/or unique sources infected humans vs. animals [209]. Mather et al. emphasized the importance of integrating veterinary and clinical data to make evidence-based judgments concerning the sources of AMR infections.

### Direct evidence of human health impact of beef antimicrobial usage

Linking on-farm antimicrobial use to human infection is difficult. While antimicrobial usage evidently selects for drug-resistant organisms, there is a gap in knowledge connecting usage to the flow of AMR determinants from the bovine microbiota to outbreaks of human AMR diseases. To bridge this gap, a number of studies compared outbreak clinical isolates to animal isolates taken at similar times from nearby locations [210–212]. Typically, isolates were examined for similar AMR/genetic profiles, and if identical, this provided some evidence of the AMR outbreak source. Direct links to specific antimicrobial usage is rarely identified for outbreaks. A caveat of many studies is that transfer is assumed to be from cattle to humans, or remains unknown. Several AMR *E. coli* and *Salmonella* outbreaks have been associated with beef [213–215], but there are few examples where those AMR determinants have been traced back to AMR bacteria in cattle [210]. This reinforces the need for greater integration of human and veterinary data. For beef production, tracing the source of an AMR outbreak is complicated by system complexity, herd movement, and lack of industry motivation. And although beef production is a major industry, more focus has been on the human health impact of AMR transfer in dairy cattle, and in the swine and poultry industries (reviewed in [214]). Dairy-related outbreaks may be easier to document because the source animal population is maintained, whereas the beef, swine, and poultry populations are consumed. Selected examples of outbreaks and human health threats posed by bovine AMR bacteria are listed in Table 2. These demonstrate that the most convincing molecular and epidemiological AMR links are found when the infected human is directly connected to the animal population on farms or via farm workers [211, 216, 217]. Direct exposure to livestock is a known risk factor for zoonotic transmission (reviewed in [218]).

**Table 2:** Selected examples of cattle-related AMR human health threats

Source	Bacterial species	Human outbreak	AMR profile	Mechanism	Notes	Study
Calves	*S. enterica* serotype Typhimurium	Veterinarian’s child	Ampicillin, chloramphenicol, tetracycline, sulfisoxazole, kanamycin, streptomycin, cephalothin, ceftriaxone and ceftiofur, aztreonam, cefoxitin, gentamicin, and tobramycin	Ceftriaxone resistance conferred by *bla*_CMY-2_ on a conjugable plasmid	An isolated, domestically acquired case requiring hospitalization. Failure of ampicillin and sulbactam therapy, but recovery with amoxicillin/clavulanate. Direct molecular evidence linking MDR isolates from herds treated by the patient’s father	[216]
Cattle, Sheep	MRSA ST130	Two farmers	Cefoxitin and penicillin	Resistance conferred by *mec*C (*mec*A homologue), SCC*mec* type XI [220]	Direct transfer of *mec*C-MRSA from cattle and sheep to humans resulting in wound infections	[211]
Veal calves	MRSA ST398	Asymptomatic carriage by farm employees	Methicillin and others	Resistance conferred by mecA, SCC*mec* not stated	Asymptomatic human MRSA carriage rates associated with prevalence in calves and frequency of animal contact. MRSA carriage in calves associated with antimicrobial use	[217]
Cattle, Swine	*E. coli*, *Salmonella*	Potential, sporadic transmission to humans	Ceftriaxone, with high-levels of co-resistance to chloramphenicols, tetracycline, sulfisoxazole, streptomycin, gentamicin, tobramycin, and ciprofloxacin	3^rd^ generation cephalosporin resistance conferred by plasmid-born *bla*_CMY-2_	Potential transfer of *bla*_CMY-2_ plasmids between *E. coli* and *Salmonella*. Close relationship between *bla*_CMY-2_ plasmids in *E. coli* found in bovines and humans	[210]
Ground beef, possibly from dairy cows	*S. enterica* serotype Typhimurium DT104	Large clustered human outbreak	Ampicillin, chloramphenicol, streptomycin, sulfemethoxazole, and tetracycline (R-type ACSSuT)	MDR genes potentially encoded on *Salmonella* genomic island 1	Multi-state outbreak, potentially affecting >2200 people. Severe illness, with a high proportion of patients receiving intravenous rehydration and requiring hospitalization	[213]

## Conclusions & future focus

As in most environments, AMR determinants exist ubiquitously in the beef production biome, regardless of antimicrobial exposure. Nevertheless, the use of antimicrobials for bovine welfare and growth promotion contributes selective pressure that increases the abundance of AMR genes and their host bacteria, and promotes the genesis and dissemination of MDR organisms. The presence or absence of connections between AMR in bovine microbial populations to human health threats are likely to become clearer with the increasing application of whole-genome sequencing and metagenomic resistomics. The role of MGEs in AMR propagation is likely to be an important focus for understanding the impact of veterinary antimicrobials. Future investigations may validate mitigation strategies, such as the separation of antimicrobials for use in beef cattle from those used in humans. Proper and judicious use of antimicrobials will help prolong the usefulness of both clinical and veterinary antimicrobials, but ever-increasing usage of antimicrobials in food-animal production suggests that microbes will only continue to acquire resistance. Of particular concern for cattle are the MDR BRD agents: in the future, respiratory infections may become untreatable with current antimicrobials. On a positive note, several studies reveal that adequate hygiene and appropriate treatment at slaughterhouse and wastewater treatment facilities are efficacious at reducing or eliminating transmission of AMR organisms and genes. Thus, such procedures and facilities should be explored further, and promoted in deficient areas of food-animal production.

## Methods

### Literature search

The literature search was conducted from January to March 2016 via Google Scholar and PubMed. Recent (2012-present) studies that described AMR or usage in context with beef production, bovine pathogens, commensal bacteria, metagenomics, the resistome, and cattle were included. Older reports, or studies referring to dairy operations were excluded, except for where beef production information was sparse.

### Comparison of most frequently reported AMR in bovine pathogens

A literature search was conducted for AMR in bovine pathogens. Journal articles ([30–88], 2000-present) were collected if the AMR data was presented in a format conducive to comparison. Reports that determined the percentage of resistant isolates in a larger collection of isolates were considered. Reports were not considered if the collection of isolates had been pre-screened or enriched for resistance to any antimicrobial. The percent resistance value (i.e. number of resistant isolates compared to the total number of isolates) for each antimicrobial tested and for each strain was recorded. Journal articles that did not provide resistant, intermediate, or susceptible determinations were excluded. Intermediate resistance was not included in the percent resistance. For several antimicrobials/species, the percent resistance was given by author-determined values; in many cases, resistance was determined according to standardized interpretive criteria. The median of the percent of resistant isolates was calculated, and the resulting median value is proportional to the diameter of each circle in Fig. 2.

## Abbreviations

AGP: Antimicrobial growth promoter
AMR: Antimicrobial resistant/resistance
ARDB: Antimicrobial resistance gene database
BRD: Bovine respiratory disease
CARD: The comprehensive antibiotic resistance database
CIPARS: Canadian integrated program for antimicrobial resistance surveillance
CoNS: Coagulase-negative *Staphylococcus*
EHEC: Enterohaemorrhagic *E. coli*
ESBL: Extended spectrum β-lactamase
ESVAC: European surveillance of veterinary antimicrobial consumption
FDA: Food and Drug Administration
HGT: Horizontal gene transfer
ICE: Integrative and conjugative element
MDR: Multi-drug resistance
MGE: Mobile genetic element
MG-RAST: Metagenomics rapid annotation using subsystem technology
NARMS: National antimicrobial resistance monitoring system
PCU: Population correction unit
STEC: Shiga toxin-producing *E. coli*
UK-VARSS: UK veterinary antibiotic resistance and sales surveillance
